# Initial dosage optimization of olanzapine in patients with bipolar disorder based on model-informed precision dosing: a study from the real world

**DOI:** 10.3389/fphar.2024.1444169

**Published:** 2024-08-21

**Authors:** Xiao Chen, Ke Hu, Hao-Zhe Shi, Liang Chen, Yi-Jia Zhang, Su-Mei He, Cun Zhang, Dong-Dong Wang

**Affiliations:** ^1^ School of Nursing, Xuzhou Medical University, Xuzhou, Jiangsu, China; ^2^ Jiangsu Key Laboratory of New Drug Research and Clinical Pharmacy and School of Pharmacy, Xuzhou Medical University, Xuzhou, Jiangsu, China; ^3^ Department of Pharmacy, Suzhou Research Center of Medical School, Suzhou Hospital, Affiliated Hospital of Medical School, Nanjing University, Suzhou, Jiangsu, China; ^4^ Department of Pharmacy, Xuzhou Oriental Hospital Affiliated to Xuzhou Medical University, Xuzhou, Jiangsu, China

**Keywords:** initial dosage optimization, olanzapine, bipolar disorder, model-informed precision dosing, real-world study

## Abstract

**Objectives:**

Olanzapine is used for treating bipolar disorder (BPD); however, the optimal initial dosing regimen is unclear. The present study aimed to investigate the optimal olanzapine initial dosage in patients with BPD via model-informed precision dosing (MIPD) based on a real-world study.

**Methods:**

Thirty-nine patients with BPD from the real-world study were collected to construct the MIPD model.

**Results:**

Weight, combined used quetiapine influenced olanzapine clearances in patients with BPD, where the clearance rates were 0.152:1 in patients with or without quetiapine under the same weight. We simulated olanzapine doses once a day or twice a day, of which twice a day was optimal. Without quetiapine, for twice-a-day olanzapine doses, 0.80, 0.70, and 0.60 mg/kg/day were suitable for 40- to 56-kg BPD patients, 56- to 74-kg BPD patients, and 74- to 100-kg BPD patients, respectively. With quetiapine, for twice-a-day olanzapine doses, 0.05 mg/kg/day was suitable for 40- to 100-kg BPD patients.

**Conclusion:**

This study was the first to investigate the optimal olanzapine initial dosage in patients with BPD via MIPD based on a real-world study, providing clinical reference for the precision medication of olanzapine in BPD patients.

## 1 Introduction

Bipolar disorder (BPD) is a common psychiatric disorder, affecting more than 1% of the population (Grande et al., 2016). In contrast to other affective disorders, BPD is characterized by recurrent alternating manic or hypomanic episodes and depressive episodes. The World Health Organization (WHO) states that approximately 60 million people suffer from BPD (Dilsaver, 2011). With the development of society, the prevalence rate of BPD increases with each passing year. Currently, BPD ranks 17th among all diseases in the world and is the main cause of disability (Vigo et al., 2016). According to the World Mental Health Survey Report, the lifetime prevalence of BPD is estimated to be 2.4%, and the 12-month prevalence is 1.5% (Merikangas et al., 2011). BPD has become the most heavily insured mental illness in the United States alone. The economic burden is estimated at $30.7 billion in direct medical costs and $120.3 billion in indirect medical costs (Dilsaver, 2011; McIntyre et al., 2020). BPD usually causes more pronounced dysfunction and has a greater impact on the quality of life than other mood disorders.

The current treatment of BPD is mainly drug therapy, that is, second-generation antipsychotics, anticonvulsants, and lithium salt treatment (Lopez-Munoz et al., 2018). In addition, studies have shown that olanzapine also can be used for the treatment of BPD (Novick et al., 2017; Uguz and Kirkas, 2021; Shoshina et al., 2022), where olanzapine is well absorbed and probably needs 6 h after an oral dose to reach the maximum plasma concentration, of which 93% olanzapine is bound primarily to albumin and α1-acid glycoproteins (Callaghan et al., 1999; Mauri et al., 2018; Mao et al., 2023). In addition, olanzapine is mainly metabolized by cytochrome P450 (CYP) 1A2, CYP2D6, and uridine diphosphate glucuronosyltransferase (UGT) 1A4, UGT2B10, and only 7% dose is excreted unchanged through urine (Sheehan et al., 2010; Soderberg and Dahl, 2013; Mao et al., 2023).

The curative effect of olanzapine is closely related to the blood drug concentration; a higher concentration is associated with adverse reactions and low levels with poor efficacy (Gex-Fabry et al., 2003; Ascher-Svanum et al., 2010; Chen et al., 2011; Kang et al., 2022; Fekete et al., 2023). Usually, in the clinical treatment of olanzapine, therapeutic drug monitoring (TDM) is applied for adjusting the next dose of olanzapine. However, there are no monitoring values for olanzapine therapy for initial administration. Therefore, how to formulate the optimal initial drug administration program is an urgent clinical problem. Xiao et al. (2022) reported the population pharmacokinetics and dosing optimization of olanzapine in Chinese pediatric patients based on the impact of sex and concomitant valproate on clearance. Zhang et al. (2024) reported the effects of aripiprazole on olanzapine population pharmacokinetics and initial dosage optimization in schizophrenic patients. Thus, the present study aimed to investigate the optimal olanzapine initial dosage in patients with BPD via model-informed precision dosing (MIPD) based on a real-world study.

## 2 Methods

### 2.1 Data collection

Patients with BPD who were treated with olanzapine from *Xuzhou Oriental Hospital Affiliated to Xuzhou Medical University* between July 2020 and August 2023 were included for the analysis (ethics approval no. 20220725011). The collected information mainly included olanzapine concentrations, physiological and biochemical indexes, and concomitant drugs.

### 2.2 Modeling

NONMEM software (edition 7, ICON Development Solutions, Ellicott City, MD, United States) was used for establishing an olanzapine population pharmacokinetic model of BPD patients, where apparent oral clearance (CL/F), volume of distribution (V/F), and absorption rate constant [Ka, fixed at 0.861/h (Sun et al., 2021)] were taken into consideration.

Formula 1 assessed inter-individual variabilities:

$$Q_{i}=\text{TV}\left(Q\right)\times \exp\left(\eta_{i}\right).$$ (1)

Here, Q_i_ represents the individual parameter, TV(Q) represents the typical individual parameter, and η_i_ represents the symmetrical distribution (0, ω^2^).

Formula 2 assessed random residual variabilities:

$$P_{i}=R_{i}+R_{i*}\varepsilon_{1}+\varepsilon_{2}.$$ (2)

Here, P_i_ represents the observed concentration, R_i_ represents the individual predicted concentration, and ε_n_ represents the symmetrical distribution (0, σ^2^).

Formula 3 assessed the weight relationship:

$$T_{i}=T_{std}\times {\left(U_{i}/U_{std}\right)}^{w}.$$ (3)

Here, T_i_ is the ith individual parameter, U_i_ is the ith individual weight, U_std_ represents 70 kg, and T_std_ represents the typical individual parameter. W represents the allometric coefficient (CL/F was 0.75 and V/F was 1 [Anderson and Holford, 2008]).

Formulas 4, 5 described the pharmacokinetic parameters between continuous covariates and categorical covariates:

Qi=TVQ×CoviCovmθ, (4)

$$Q_{i}=\text{TV}\left(Q\right)\times \left(1+\theta \times {\text{Cov}}_{i}\right).$$ (5)

Here, Q_i_ represents the individual parameter, TV(Q) represents the typical individual parameter, and θ needs to be evaluated. Cov_i_ represents the ith individual covariate and Cov_m_ represents the population covariate median. The covariate model was built up in a stepwise manner. Objective function value reduction > 6.63 (*p* < 0.01) and increase > 10.8 (*p* < 0.001) were selected as the inclusion and exclusion standards, respectively.

### 2.3 Model validation

Observations vs. population predictions, observations vs. individual predictions, absolute value of weighted residuals of the individual (│iWRES│) vs. individual predictions, weighted residuals vs. time, density vs. weighted residuals, quantiles of weighted residuals vs. quantiles of normal, visual predictive check (VPC) of the model, and individual plot were used for evaluating the final model. Furthermore, the median and 2.5th–97.5th percentiles of bootstrap (n = 1,000) were used for comparing with the final model parameters.

### 2.4 Simulation

Initial dosage optimization of olanzapine in patients with BPD was done via Monte Carlo simulation, in which the therapeutic range of olanzapine in patients with BPD was 20–80 ng/mL (Ding et al., 2022). In the final population pharmacokinetic model, weight and combined used quetiapine influenced olanzapine clearances in patients with BPD. Thus, on the basis of whether quetiapine was used in combination, and once-a-day or twice-a-day olanzapine doses, we simulated four different situations, where each situation contained 1,000 virtual patients with BPD, 11 doses (0.05, 0.10, 0.20, 0.30, 0.40, 0.50, 0.60, 0.70, 0.80, 0.90, and 1.00 mg/kg/day) for 7 weight groups (40, 50, 60, 70, 80, 90, and 100 kg), respectively, where the probability of achieving the target concentration was selected as the evaluation criterion. The twice-a-day olanzapine doses were split evenly into two dosages a day.

## 3 Results

### 3.1 Patient information

A total of 39 patients with BPD from the real-world study, including 25 men and 14 women, whose ages ranged from 14.99 to 73.09 years and weights ranged from 45.00 to 85.00 kg, participated in the study. Patient information and drug use are shown in Tables 1, 2, respectively

**TABLE 1:** Demographic data of patients with bipolar disorder (n = 39).

Characteristic	Mean ± SD	Median (range)
Gender (male/female)	25/14	—
Age (years)	43.49 ± 12.48	43.09 (14.99–73.09)
Weight (kg)	70.01 ± 8.23	72.00 (45.00–85.00)
Albumin (g/L)	40.36 ± 3.41	40.20 (33.30–48.60)
Globulin (g/L)	25.90 ± 3.79	26.00 (17.10–33.80)
Alanine transaminase (IU/L)	25.58 ± 20.24	18.50 (7.00–108.00)
Aspartate transaminase (IU/L)	21.69 ± 7.87	19.00 (10.00–46.00)
Creatinine (μmol/L)	72.83 ± 23.04	68.50 (30.00–138.00)
Urea (mmol/L)	4.69 ± 2.12	4.27 (2.32–12.30)
Total protein (g/L)	66.38 ± 5.29	66.60 (51.90–75.90)
Total cholesterol (mmol/L)	4.59 ± 1.17	4.37 (2.61–7.29)
Triglyceride (mmol/L)	1.84 ± 1.11	1.55 (0.50–6.87)
Direct bilirubin (μmol/L)	2.73 ± 1.14	2.70 (0.60–6.60)
Total bilirubin (μmol/L)	7.85 ± 2.83	7.05 (3.60–16.00)
Hematocrit (%)	39.82 ± 2.79	39.50 (34.40–46.40)
Hemoglobin (g/L)	131.85 ± 9.74	131.00 (111.00–155.00)
Mean corpuscular hemoglobin (pg)	30.81 ± 1.47	31.25 (24.90–33.30)
Mean corpuscular hemoglobin concentration (g/L)	331.13 ± 10.02	331.00 (307.00–361.00)

**TABLE 2:** Drug combination in patients with bipolar disorder (n = 39).

Drug	Category	N	Drug	Category	N
Atorvastatin calcium tablets	0	36	Haloperidol injection	0	37
	1	3		1	2
Alprazolam tablets	0	35	Lorazepam tablets	0	29
	1	4		1	10
Benzhexol hydrochloride tablets	0	37	Omeprazole enteric-coated capsules	0	37
	1	2		1	2
Bezafibrate	0	37	Quetiapine fumarate tablets	0	37
	1	2		1	2
Buspirone hydrochloride tablets	0	37	Risperidone tablets	0	37
	1	2		1	2
Clonazepam tablets	0	37	Sertraline hydrochloride tablets	0	37
	1	2		1	2
Clozapine tablets	0	35	Valsartan capsules	0	37
	1	4		1	2
Escitalopram oxalate tablets	0	36	Zopiclone tablets	0	33
	1	3		1	6

Category: 0, without drug; 1, with drug; N, number of patients.

### 3.2 Modeling

Weight and combined used quetiapine influenced olanzapine clearances in patients with BPD, which were shown in Formulas 6, 7:

CLF=18.5×weight700.75×1−0.848×QUE, (6)

$$V/F=106\times \left(\text{weight }/70\right).$$ (7)

Here, QUE denotes quetiapine. When patients took quetiapine, QUE was 1; otherwise, QUE was 0.

### 3.3 Evaluation

**FIGURE 1:**
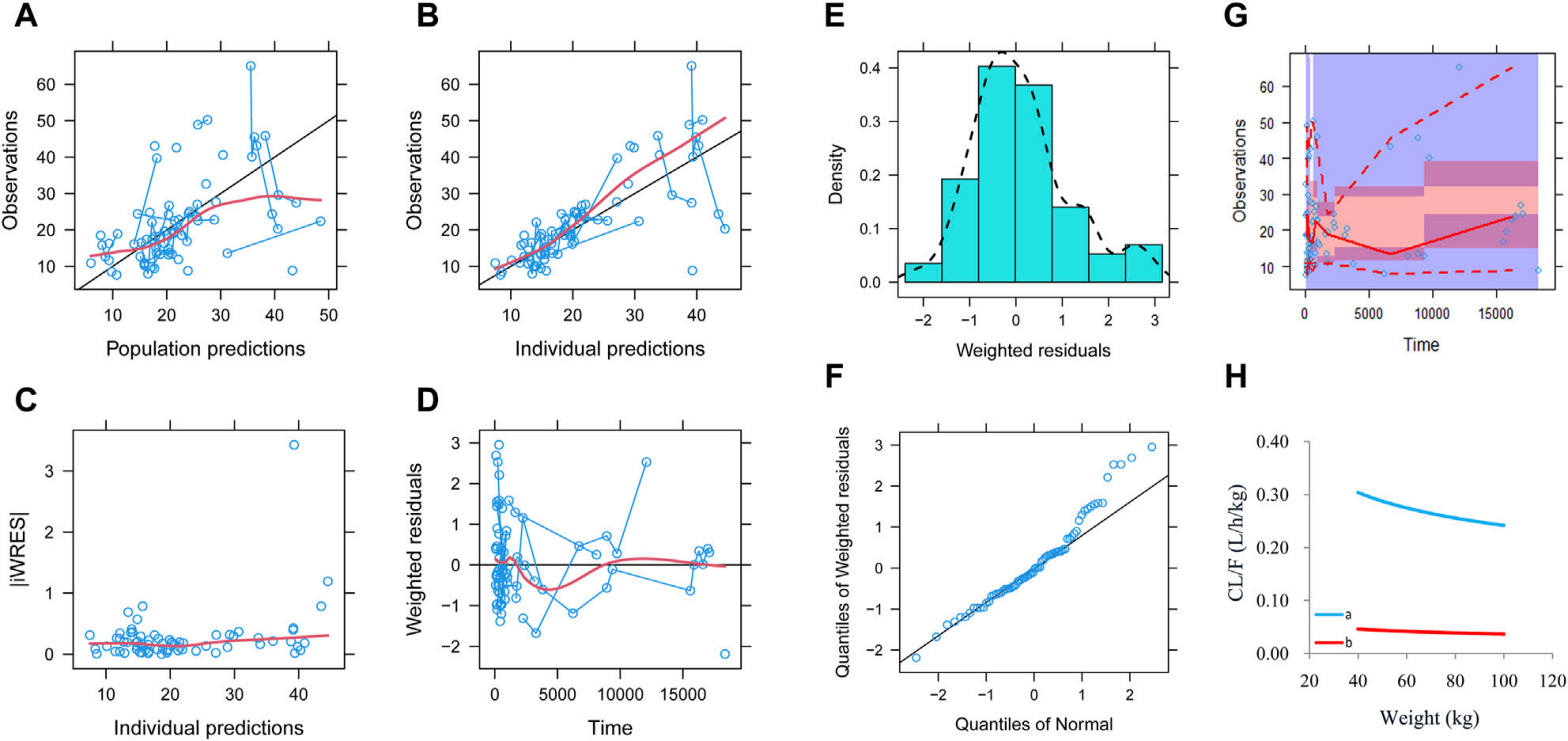
Model evaluation. **(A)** Observations vs. population predictions. **(B)** Observations vs. individual predictions. **(C)** Absolute value of weighted residuals of the individual (│iWRES│) vs. individual predictions. **(D)** Weighted residuals vs. time. **(E)** Density vs. weighted residuals. **(F)** Quantiles of weighted residuals vs. quantiles of normal. **(G)** Visual predictive check (VPC) of the model. **(H)** Olanzapine clearance: (a) without quetiapine and (b) with quetiapine.

**FIGURE 2:**
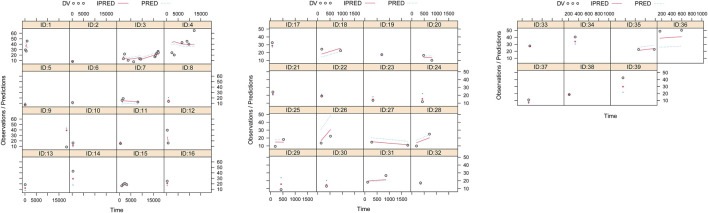
Individual plot. ID, patient ID number; DV, measured concentration value; IPRED, individual predictive value; PRED, population predictive value.

Observations vs. population predictions, observations vs. individual predictions,│iWRES│ vs. individual predictions, weighted residuals vs. time, density vs. weighted residuals, quantiles of weighted residuals vs. quantiles of normal, and VPC of the model are shown in Figures 1A–G, indicating that the final model predicted well. The clearance rates were 0.152:1 in patients with or without quetiapine under the same weight, as shown in Figure 1H. The individual plot is shown in Figure 2, and the bootstrap results are shown in Table 3. The above results showed that the model is robust and reliable.

**TABLE 3:** Parameter estimates and bootstrap validation in patients with bipolar disorder.

Parameter	Estimate	RSE (%)/shrinkage [%]	Bootstrap		Bias (%)
			Median	95% confidence interval	
CL/F (L/h)	18.5	(16%)	18.9	[12.6, 26.2]	2.16
V/F (L)	106	(32%)	113	[52, 282]	6.60
Ka (h^−1^)	0.861 (fixed)	—	—	—	—
θ_QUE_	−0.848	(4%)	−0.836	[−0.896, −0.262]	−1.42
ω_CL/F_	18.4%	[20%]	18.5%	[9.2%, 36.9%]	0.54
σ_1_	30.7%	[15%]	27.1%	[13.3%, 37.8%]	−11.73
σ_2_ (ng/mL)	1.764	[15%]	2.298	[0.526, 4.261]	30.27

95% confidential interval was displayed as the 2.5th–97.5th percentile of bootstrap estimates. CL/F, apparent oral clearance (L/h); V/F, apparent volume of distribution (L); Ka, absorption rate constant (h^−1^); θ_QUE_, coefficient of quetiapine; ω_CL/F_, inter-individual variability of CL/F; σ_1_, residual variability, proportional error; σ_2_, residual variability, additive error; bias, prediction error, bias = (median-estimate)/estimate ×100%.

### 3.4 Simulation

**FIGURE 3:**
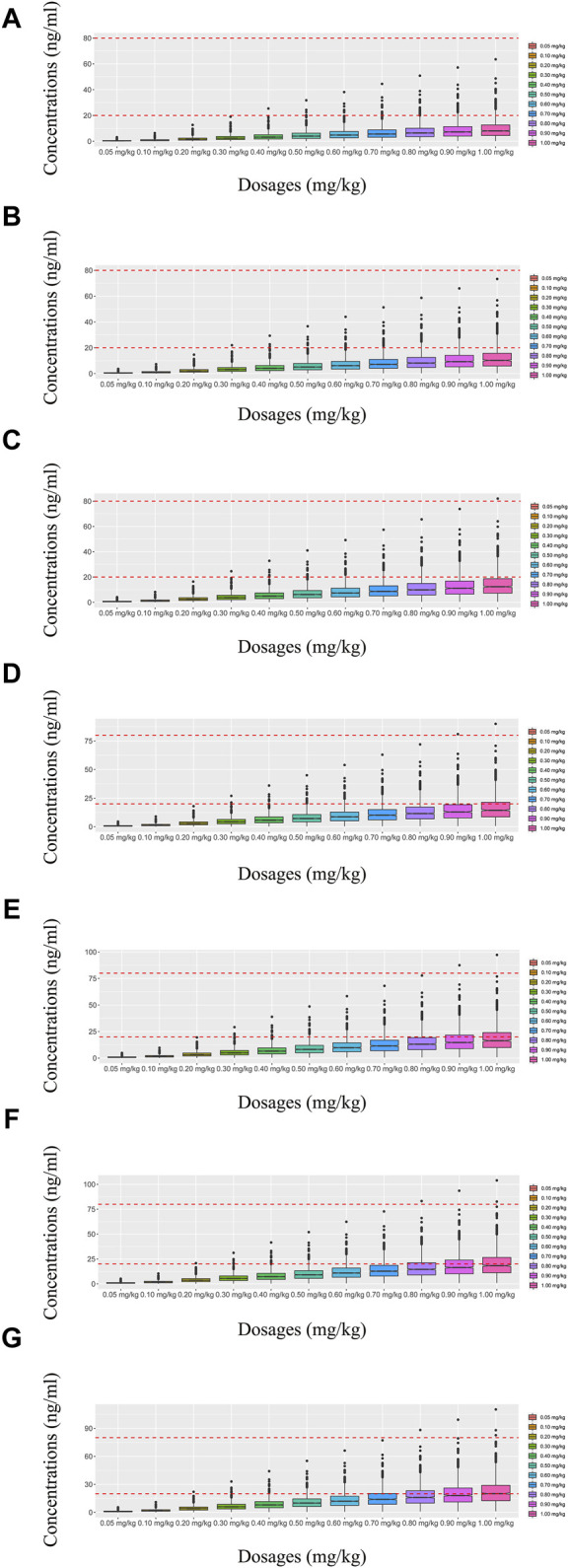
Simulated olanzapine concentrations of once-daily olanzapine administration dosages without quetiapine. **(A)** BPD patients of 40 kg, **(B)** BPD patients of 50 kg, **(C)** BPD patients of 60 kg, **(D)** BPD patients of 70 kg, **(E)** BPD patients of 80 kg, **(F)** BPD patients of 90 kg, and **(G)** BPD patients of 100 kg. The lower and upper red dashed lines denote 20 and 80 ng/mL, respectively.

In our model, we simulated four different situations: 1) once-a-day olanzapine doses without quetiapine, 2) twice-a-day olanzapine doses without quetiapine, 3) once-a-day olanzapine doses with quetiapine, and 4) twice-a-day olanzapine doses with quetiapine. Figures 3A–G, 6A–G show the concentration–dosage plots for BPD patients of 40 kg–100 kg. Figure 7 shows the probabilities of achieving the target concentrations, where Figures 7A–D represent once-a-day olanzapine doses without quetiapine, twice-a-day olanzapine doses without quetiapine, once-a-day olanzapine doses with quetiapine, and twice-a-day olanzapine doses with quetiapine, respectively.

**FIGURE 4:**
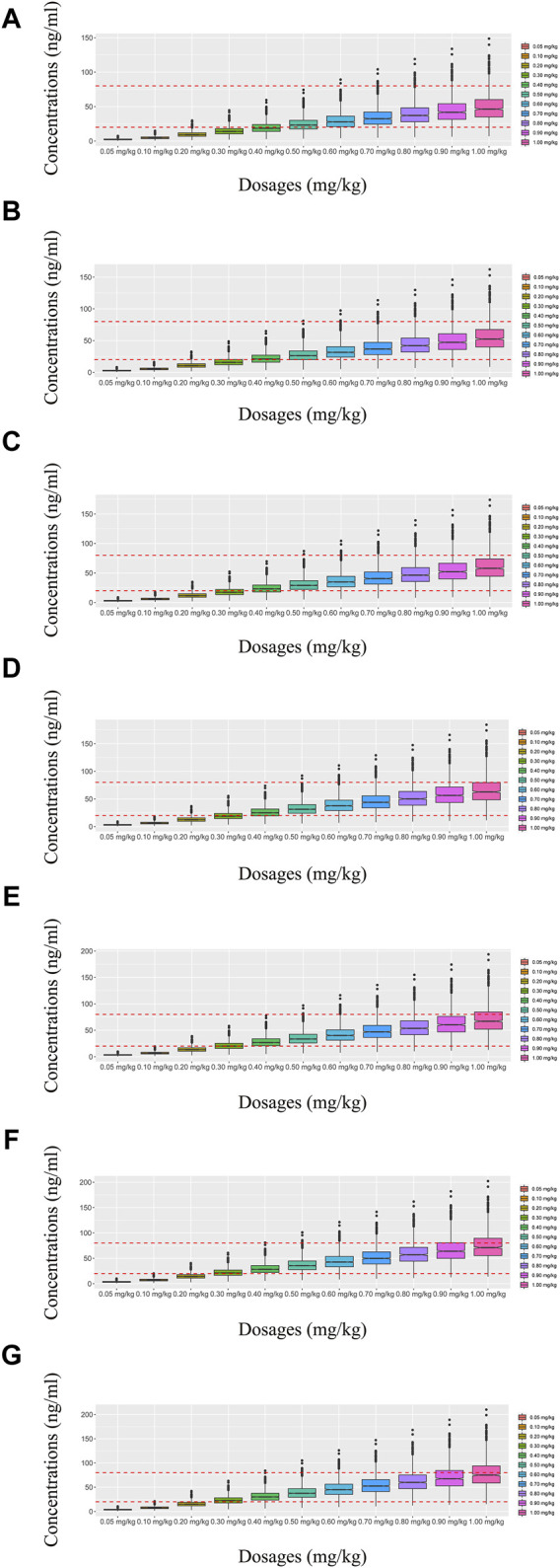
Simulated olanzapine concentrations of twice-daily olanzapine administration dosages without quetiapine. **(A)** BPD patients of 40 kg, **(B)** BPD patients of 50 kg, **(C)** BPD patients of 60 kg, **(D)** BPD patients of 70 kg, **(E)** BPD patients of 80 kg, **(F)** BPD patients of 90 kg, and **(G)** BPD patients of 100 kg. The lower and upper red dashed lines denote 20 and 80 ng/mL, respectively.

**FIGURE 5:**
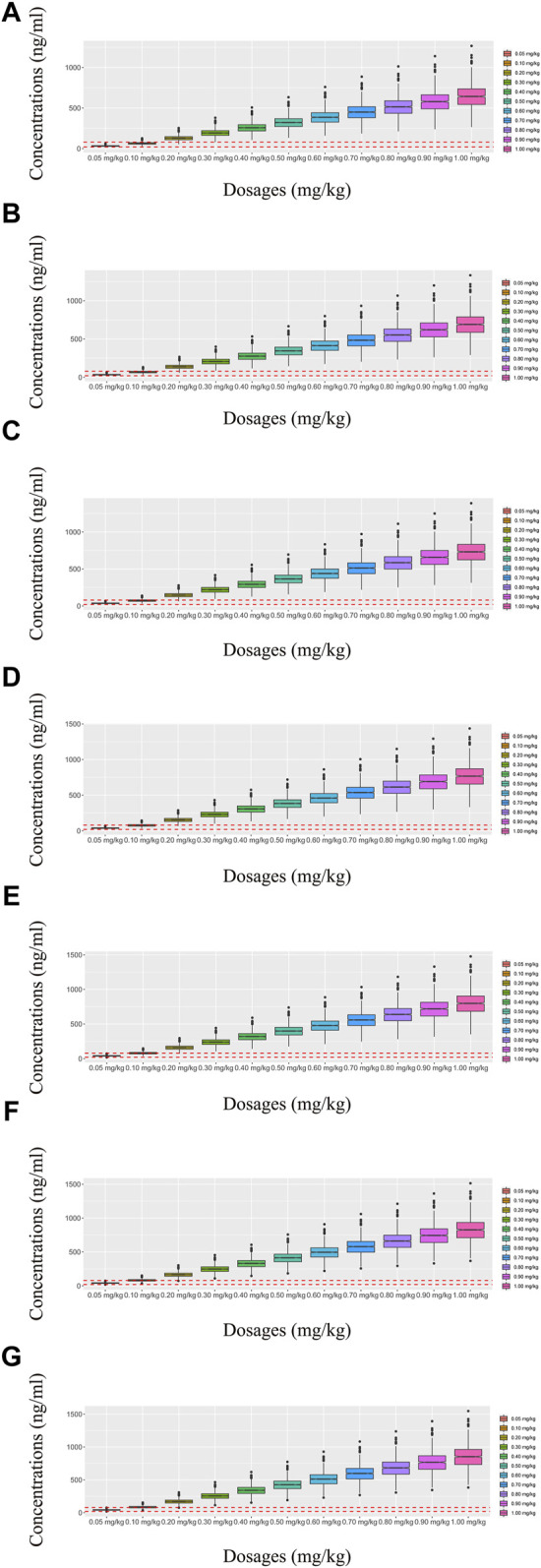
Simulated olanzapine concentrations of once-daily olanzapine administration dosages with quetiapine. **(A)** BPD patients of 40 kg, **(B)** BPD patients of 50 kg, **(C)** BPD patients of 60 kg, **(D)** BPD patients of 70 kg, **(E)** BPD patients of 80 kg, **(F)** BPD patients of 90 kg, and **(G)** BPD patients of 100 kg. The lower and upper red dashed lines denote 20 and 80 ng/mL, respectively.

**FIGURE 6:**
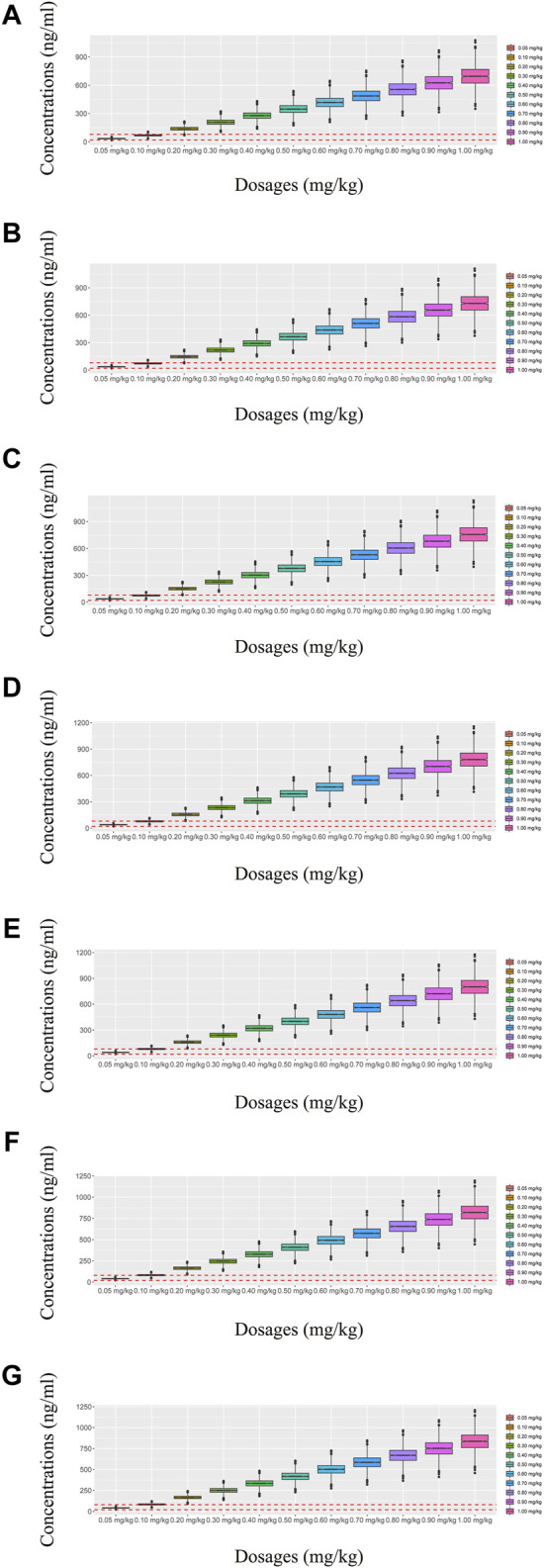
Simulated olanzapine concentrations of twice-daily olanzapine administration dosages with quetiapine. **(A)** BPD patients of 40 kg, **(B)** BPD patients of 50 kg, **(C)** BPD patients of 60 kg, **(D)** BPD patients of 70 kg, **(E)** BPD patients of 80 kg, **(F)** BPD patients of 90 kg, and **(G)** BPD patients of 100 kg. The lower and upper red dashed lines denote 20 and 80 ng/mL, respectively.

**FIGURE 7:**
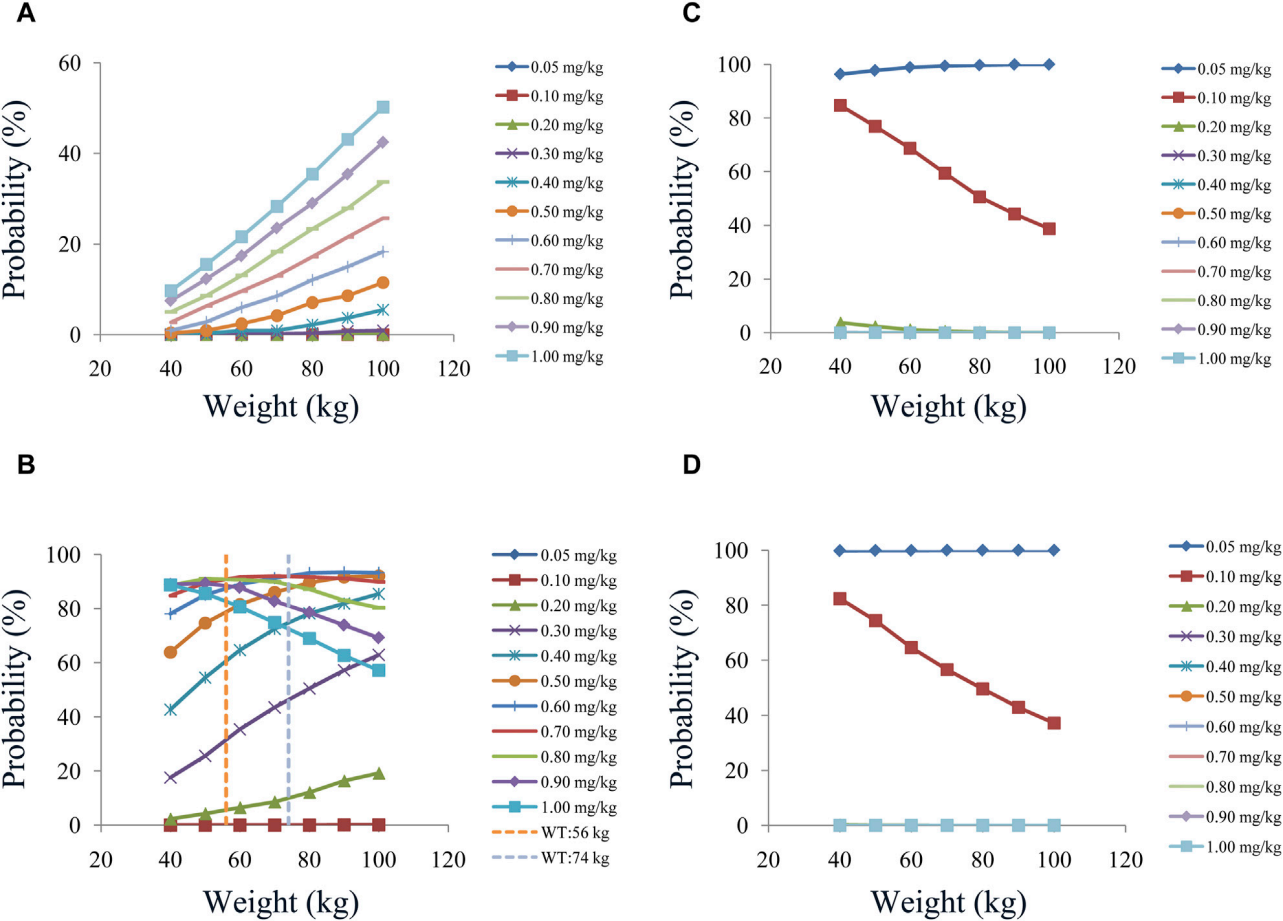
Probabilities of achieving the therapeutic window: **(A)** once-daily olanzapine administration dosages without quetiapine, **(B)** twice-daily olanzapine administration dosages without quetiapine, **(C)** once-daily olanzapine administration dosages with quetiapine, and **(D)** twice-daily olanzapine administration dosages with quetiapine.

According to simulation results, Table 4 shows the optimal olanzapine initial dosages for patients with BPD. Without quetiapine, for once-a-day olanzapine doses, the probability of achieving the target concentrations from all dosages (0.05–1.00 mg/kg/day) was less than 50.2%. For twice-a-day olanzapine doses, 0.80, 0.70, and 0.60 mg/kg/day were recommended for 40- to 56-kg BPD patients, 56- to 74-kg BPD patients, and 74- to 100-kg BPD patients, respectively; simultaneously, the probabilities of achieving the target concentrations for the dosages of 0.80, 0.70, and 0.60 mg/kg/day were 88.6%–91.0%, 91.0%–92.1%, and 92.0%–93.5%, respectively. With quetiapine, for once-a-day olanzapine dose, 0.05 mg/kg/day was recommended for 40- to 100-kg BPD patients, and the probability of achieving the target concentrations was 96.3%–99.9%. For the twice-a-day olanzapine dose, 0.05 mg/kg/day was recommended for 40- to 100-kg BPD patients, and the probability of achieving the target concentrations was 99.8%–100.0%.

**TABLE 4:** Initial dosage recommendation of olanzapine in bipolar disorder patients without or with quetiapine.

Without quetiapine			With quetiapine		
Once a day			Once a day		
Body weight (kg)	Dose (mg/kg/day)	Probability to achieve the target concentrations (%)	Body weight (kg)	Dose (mg/kg/day)	Probability to achieve the target concentrations (%)
[40–100]	0.05–1.00	all ≤50.2	[40–100]	0.05	96.3–99.9

Split evenly into two doses a day			Split evenly into two doses a day		
Body weight (kg)	Dose (mg/kg/day)	Probability to achieve the target concentrations (%)	Body weight (kg)	Dose (mg/kg/day)	Probability to achieve the target concentrations (%)
[40–56]	0.80	88.6–91.0	[40–100]	0.05	99.8–100.0
[56–74]	0.70	91.0–92.1			
[74–100]	0.60	92.0–93.5			

## 4 Discussion

According to the latest psychiatric drug monitoring guidelines issued by the German Association of Neuropsychopharmacology and Pharmacopsychiatry (AGNP) (Hiemke et al., 2018), olanzapine is a “class I (highly recommended)” drug for TDM. The steady-state plasma concentration of olanzapine is correlated with its efficacy/adverse reactions. The AGNP consensus guidelines also emphasize that the interpretation of the measured drug concentrations needs to take into account pharmacokinetic variations that should be considered when optimizing olanzapine administration regimens in clinical practice. Therefore, it is urgent to solve the individualized administration of olanzapine in BPD patients.

In this study, we selected the most commonly used population pharmacokinetic model from MIPD to optimize the initial dosage of olanzapine in patients with BPD; this approach has been reported in previous studies of individualized therapy of other drugs (Jaruratanasirikul et al., 2019; Gil Candel et al., 2020; Nguyen et al., 2021; Chung and Seto, 2023; Salcedo-Mingoarranz et al., 2023). Therefore, the present study aimed to investigate the optimal olanzapine initial dosage in patients with BPD via a population pharmacokinetic model based on the real-world study.

In a previous study (Sun et al., 2021), the final population pharmacokinetic model for olanzapine featured a two-compartment disposition model with a lag time for absorption. In the current study, a one-compartment model was selected as the final model. This was mainly due to the difference in datasets affecting our choice of structural models. For example, in the previous study (Sun et al., 2021), olanzapine data were obtained from intense sampling and sparse sampling, and in the current study, olanzapine data were obtained from clinical sparse sampling of TDM.

In the present study, 39 patients with BPD from the real-world study, including 25 men and 14 women, participated in the study. Demographic data of patients with BPD and drug combinations were used to analyze potential influencing factors. In the final model, the olanzapine typical value of CL/F was 18.5 L/h in BPD patients, which was similar to the olanzapine typical value of CL/F (27.6 L/h) in schizophrenic patients (Zhang et al., 2024). In addition, weight and combined used quetiapine influenced olanzapine clearances in patients with BPD, where the clearance rates were 0.152:1 in patients with or without quetiapine under the same weight. The drug interaction between quetiapine and olanzapine was mainly due to quetiapine, and its metabolites could inhibit CYP1A2 and CYP2D6, whereas olanzapine was metabolized through CYP1A2 and CYP2D6 (Carrillo et al., 2003; Shirley et al., 2003; Bakken et al., 2012), and quetiapine inhibited the metabolism of olanzapine.

Similarly, in the drug interactions of olanzapine, Zhang et al. (2024) reported the effects of aripiprazole on olanzapine in schizophrenic patients. The previous study found that without aripiprazole, for once-daily olanzapine administration dosages, 0.6 and 0.5 mg/kg/day were recommended for 40- to 70-kg schizophrenic patients and 70- to 100-kg schizophrenic patients, respectively; for twice-daily olanzapine administration dosages, 0.6 and 0.5 mg/kg/day were recommended for 40- to 60-kg schizophrenic patients and 60- to 100-kg schizophrenic patients, respectively (Zhang et al., 2024). With aripiprazole, for once-daily olanzapine administration dosages, 0.4 and 0.3 mg/kg/day were recommended for 40- to 53-kg schizophrenic patients and 53- to 100-kg schizophrenia patients, respectively; for twice-daily olanzapine administration dosages, 0.4 mg/kg/day was recommended for 40- to 100-kg schizophrenic patients (Zhang et al., 2024).

Furthermore, we simulated once-a-day or twice-a-day olanzapine doses, of which twice-a-day was optimal. Without quetiapine, for twice-a-day olanzapine doses, 0.80, 0.70, and 0.60 mg/kg/day were recommended for 40- to 56-kg BPD patients, 56- to 74-kg BPD patients, and 74- to 100-kg BPD patients, respectively. With quetiapine, for the twice-a-day olanzapine dose, 0.05 mg/kg/day was recommended for 40- to 100-kg BPD patients. In other words, there are differences in the optimal administration regimen of olanzapine in different diseases, and the optimal administration regimen should be selected clinically according to the specific disease.

There were some limitations in this study. As our olanzapine data were collected from the real-world clinical sparse trough concentrations, the ability to predict was objectively deficient. However, it did not influence the overall model and its clinical application. In the future, we will design prospective olanzapine intensive sampling sites to further carry out relevant studies. In addition, at present, there were some obstacles in realizing the precise drug delivery scheme of olanzapine products, so it was recommended to develop appropriate dosage forms in drug research and development. At the same time, further studies should be conducted to verify the safety and effectiveness of the recommended dosages.

## 5 Conclusion

This study was the first to investigate the optimal olanzapine initial dosage in patients with BPD via MIPD based on the real-world study, providing a clinical reference for the precision medication of olanzapine in BPD patients.

## Data Availability

The original contributions presented in the study are included in the article/Supplementary Material; further inquiries can be directed to the corresponding authors.
